# Chasing “Emerging” Contaminants: An Endless Journey toward Environmental Health

**DOI:** 10.1021/acs.est.3c10630

**Published:** 2024-01-19

**Authors:** Peng Gao

**Affiliations:** † Department of Environmental and Occupational Health and Department of Civil and Environmental Engineering, University of Pittsburgh, Pittsburgh, Pennsylvania 15261, United States; ‡ UPMC Hillman Cancer Center, Pittsburgh, Pennsylvania 15232, United States

**Keywords:** emerging contaminants, legacy contaminants, environmental health, green chemistry, One Health, exposome, omics technologies, regulatory frameworks

In the ever-evolving landscape of environmental science and technology, the relentless pursuit of identifying and mitigating “emerging” contaminants presents a Sisyphean task for scientists and policymakers alike. As an analytical chemist and exposure scientist, my career has been intricately woven into this unending journey. It begins anew with each industrial innovation, introducing novel chemicals into our environment, often with unforeseen consequences. This cycle, while necessary for safeguarding public and ecological health, raises an existential question within the scientific community. When, if ever, will this relentless pursuit conclude? Our current environmental narrative is predominantly characterized by the emergence of new contaminants. The process is almost formulaic. Industries introduce new substances, often as byproducts of technological advancement, (bio)transformation products, or replacements for previously regulated chemicals. Once released into the environment, these chemicals become the subject of intense scrutiny. Analytical chemists engage in a meticulous race to quantify their presence and distribution. Following suit, environmental scientists embark on understanding their fate and transport mechanisms through our ecosystems. Exposure scientists delve into deciphering potential pathways through which these chemicals interact with biological systems. Concurrently, toxicologists and epidemiologists study the potential adverse health effects, paving the way for environmental engineers and policy scientists to strategize their removal, remediation, and regulation. This cycle, a testament to our scientific rigor and commitment to environmental stewardship, has been repeated with various classes of contaminants, from the dichlorodiphenyltrichloroethane that spurred Rachel Carson’s environmental awakening in “Silent Spring” to contemporary concerns such as numerous plasticizers, flame retardants, and per- and polyfluoroalkyl substances.

Our focused chase after emerging contaminants often inadvertently sidelines an equally critical concern, the legacy contaminants. These not only include naturally occurring chemicals like trace metals and polycyclic aromatic hydrocarbons but also encompass a range of persistent organic pollutants such as dioxins, polychlorinated biphenyls, and various pesticides, recognized under the Stockholm Convention. The ramifications of these legacy substances, often exacerbated by the industrial era’s massive scale of production and technological advancement, are profound and enduring. Our focus on emerging contaminants should not eclipse the significance of these legacy substances, as they continue to persist and impact ecosystems and human health. Addressing these legacy contaminants effectively calls for robust regulatory frameworks that enforce ongoing monitoring and stricter remediation standards. Simultaneously, applying green chemistry principles can prevent future legacy issues by promoting the development of safer chemical alternatives. This dual approach ensures a comprehensive strategy for managing both new and existing environmental threats. However, today emerging contaminants often garner immediate attention and resources, potentially diverting focus from understanding and mitigating the long-term effects of these persistent environmental residents. The tendency to prioritize emerging over legacy contaminants reveals an inherent shortcoming in our current approach to environmental management. In our haste to address the immediate, we may inadvertently neglect the lingering and equally significant threats posed by legacy contaminants. This oversight can lead to a fragmented understanding of environmental and health risks, as the interactions between various contaminants, both new and old, are complex and often synergistic. The environmental impact of a chemical is dictated by not only its novelty but also many other factors such as distribution, concentration, persistence, biological effects, and ecological interactions. Hence, a more holistic approach that acknowledges the intricate interplay between emerging and legacy contaminants and their cumulative effects on environmental and human health is necessary.^1^

To transcend this perpetual cycle of chasing after emerging contaminants, we have to explore and embrace innovative methodologies and frameworks to form proactive and integrated strategies for environmental protection. Green chemistry principles present a promising avenue. By embedding environmental consciousness at the very inception of industrial processes and product design, green chemistry aims to minimize the generation and use of hazardous substances.^2^ However, currently implementing green chemistry is challenged by high development costs, a lack of specialized knowledge and resources, unsupportive regulatory frameworks, and insufficient educational focus. Overcoming these barriers requires government incentives, collaborative efforts between industry and academia, regulatory reform, and integration of green chemistry into educational curriculums, thus fostering a sustainable shift in chemical practices. If successful, these efforts can catalyze the transition toward more environmentally sustainable chemical practices. This proactive approach not only addresses the environmental impacts of new chemicals but also holds the potential for reinventing existing processes, reducing the overall environmental footprint. Another transformative approach lies in the integration of machine learning and artificial intelligence in environmental science. The predictive power of machine learning models can be harnessed to forecast the environmental and health impacts of new chemicals before their widespread use. These models, through the analysis of vast and complex data sets, can identify potential risks and guide the development of safer alternatives. Such predictive modeling is not just a tool for mitigation but a strategic instrument for prevention, offering a road map for more sustainable chemical design and use. Undoubtedly, effective regulatory frameworks are crucial for avoiding the “regrettable substitution” of deleterious chemicals. Current regulations often lack comprehensive risk assessments and life-cycle analyses, leading to the replacement of one toxic chemical with another. To mitigate this, regulatory reforms are needed to enforce stringent premarket testing and life-cycle analysis, ensuring replacement chemicals are safer and more sustainable. Incorporating a precautionary principle in chemical approvals can further safeguard against such substitutions, aligning chemical innovation with environmental protection. Moreover, the adoption of holistic health assessment frameworks such as One Health and the Exposome offers a comprehensive perspective on environmental risks.^3^ The One Health approach, recognizing the interconnectedness of human, animal, and environmental health, provides a multidimensional understanding of how contaminants affect our world. The Exposome, encompassing the totality of environmental exposures an individual encounters, extends this understanding to a personal level, highlighting the diverse and dynamic nature of environmental interactions.^4^ Together, these frameworks facilitate a more integrated and nuanced understanding of environmental impacts, transcending the limitations of a singular focus on either emerging or legacy contaminants. In addition to the development of advanced monitoring and detection technologies to measure environmental exposures, further deepening our understanding of their holistic biological impacts are omics technologies such as genomics, epigenomics, transcriptomics, proteomics, and metabolomics. These technologies offer unprecedented insights into the molecular mechanisms underpinning the biological responses to environmental exposures. By elucidating the complex interactions and mixture effects of various contaminants at a molecular level, omics approaches aid in developing more effective and targeted mitigation strategies.^5^ These technologies not only enhance our understanding of individual contaminants but also illuminate the broader picture of how mixtures of substances interact and impact biological systems.

**Figure 1:**
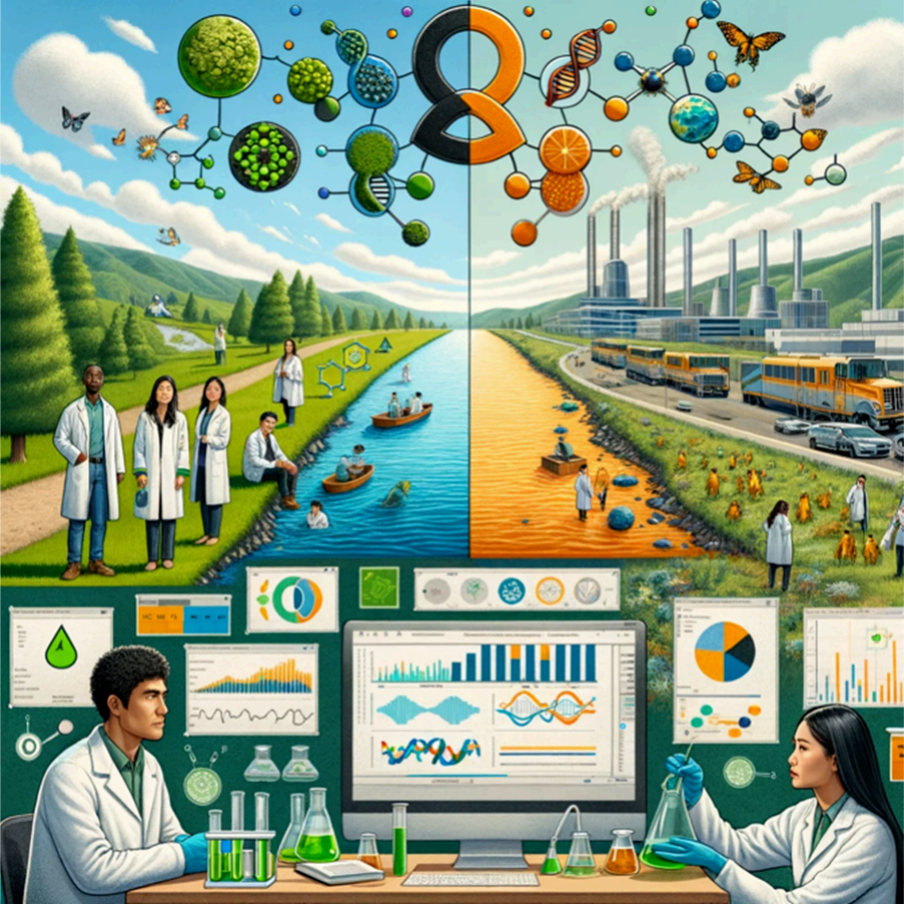
Bridging innovation and vigilance in environmental health. The figure illustrates the balance in addressing emerging and legacy contaminants. It showcases the fusion of green chemistry, machine learning, One Health, exposome, and omics in environmental science and technology. The left side depicts eco-friendly practices in natural settings, while the right highlights the study of anthropogenic contaminants, emphasizing a holistic and proactive approach.

In conclusion, addressing the spectrum of emerging and legacy contaminants demands a robust fusion of innovative strategies. Integrating green chemistry and advanced predictive technologies like machine learning is essential for preemptive risk assessment and the design of safer chemicals. Strengthening regulatory frameworks to include comprehensive risk and life-cycle analyses helps prevent the cycle of substituting one hazardous chemical for another. Holistic health frameworks such as One Health and the exposome, along with omics technologies, provide a multidimensional perspective on environmental exposure and its effects. By intertwining these innovative approaches, we endeavor to shift from a reactive to a proactive stance in environmental stewardship. This paradigm shift is vital for aligning human progress with the preservation of ecological integrity. The collective application of these strategies beckons us toward a future in which environmental and human health not only are balanced but also thrive in unison (Figure 1). Such a future, ambitious in its scope, can be attained through the concerted efforts of scientists, policymakers, industries, and communities, all dedicated to the mission of environmental science and technology.
